# One-Pot Preparation of Ratiometric Fluorescent Molecularly Imprinted Polymer Nanosensor for Sensitive and Selective Detection of 2,4-Dichlorophenoxyacetic Acid

**DOI:** 10.3390/s24155039

**Published:** 2024-08-03

**Authors:** Yuhong Cui, Xintai Li, Xianhong Wang, Yingchun Liu, Xiuli Hu, Shengli Chen, Xiongwei Qu

**Affiliations:** 1 Hebei Key Laboratory of Functional Polymers, School of Chemical Engineering and Science, Hebei University of Technology, Tianjin 300401, China; 202221501023@stu.hebut.edu.cn (Y.C.); 211879@stu.hebut.edu.cn (X.L.); huxiuli@hebut.edu.cn (X.H.); 2 Tianjin Key Laboratory of New Materials and Systems for HVAC Plumbing, Tianjin 300400, China; henliking@163.com; 3 Jinghua Plastics Co., Ltd., Langfang 065800, China; jinghuasuye@163.com

**Keywords:** molecular imprinted polymer (MIP), ratiometric fluorescent sensor, red fluorescent CdTe quantum dots (QDs), green fluorescent nitrobenzodiazole (NBD), 2,4-dichlorophenoxyacetic acid (2,4-D)

## Abstract

The development of fluorescent molecular imprinting sensors for direct, rapid, and sensitive detection of small organic molecules in aqueous systems has always presented a significant challenge in the field of detection. In this study, we successfully prepared a hydrophilic colloidal molecular imprinted polymer (MIP) with 2,4-dichlorophenoxyacetic acid (2,4-D) using a one-pot approach that incorporated polyglycerol methacrylate (PGMMA-TTC), a hydrophilic macromolecular chain transfer agent, to mediate reversible addition-fragmentation chain transfer precipitation polymerization (RAFTPP). To simplify the polymerization process while achieving ratiometric fluorescence detection, red fluorescent CdTe quantum dots (QDs) and green fluorescent nitrobenzodiazole (NBD) were introduced as fluorophores (with NBD serving as an enhancer to the template and QDs being inert). This strategy effectively eliminated background noise and significantly improved detection accuracy. Uniform-sized MIP microspheres with high surface hydrophilicity and incorporated ratiometric fluorescent labels were successfully synthesized. In aqueous systems, the hydrophilic ratio fluorescent MIP exhibited a linear response range from 0 to 25 μM for the template molecule 2,4-D with a detection limit of 0.13 μM. These results demonstrate that the ratiometric fluorescent MIP possesses excellent recognition characteristics and selectivity towards 2,4-D, thus, making it suitable for selective detection of trace amounts of pesticide 2,4-D in aqueous systems.

## 1. Introduction

Molecular imprinting, as a customizable synthetic method, plays a pivotal role in biosensors, food testing, environmental monitoring, and adsorptive separation. Among these applications, the development of fluorescence-based molecularly imprinted sensors holds significant importance [1,2,3,4,5]. The sensitivity of fluorescence can be enhanced by increasing its strong emission signal or reducing the noise of the detection system, in comparison to other optical signals such as UV and Raman [6,7]. When combined with molecular imprinting technology, it results in a fluorescent molecularly imprinted sensor that offers remarkable advantages such as enhanced sensitivity, real-time and in situ detection capabilities, rapid response time, low sample consumption, and relatively low cost [8,9,10,11,12]. The sensitive and rapid quantitative detection of small organic molecules in aqueous samples is crucial for food safety assessment and environmental analysis; however, it remains a challenge [13,14,15,16,17]. Furthermore, accurate measurement can be influenced by various factors, including the environment of the detection solution itself as well as the detection instrument used or even the fluorophore employed [5,18,19,20]. To overcome these limitations and interference factors during the measurement process, a ratiometric fluorescence technique is integrated with molecular imprinting technique using two selected fluorophores [21,22,23]. This enables quantitative determination of template concentration based on the intensity ratio between two fluorescence emission wavelengths, thereby compensating for potential interfering factors [24,25,26].

In recent years, there has been a significant upsurge in research interest regarding ratiometric fluorescent molecularly imprinted polymer (MIP) chemical sensors [27,28,29,30,31,32]. MIP technology utilizes the analyte as a template to interact with monomers or polymers, resulting in the formation of a polymeric shell that mimics a cast. Upon removal of the analyte, an empty cavity is created which retains molecular memory for the target analyte, similar to a natural antibody–antigen system [31,32]. This enables potential specific and selective detection of the analyte in complex environmental conditions at a low cost. Notably, remarkable advancements have been achieved with highly sensitive turn-off (or fluorescence quenching) and turn-on (or fluorescence enhancement) type fluorescent MIP sensors [32,33,34,35,36]. However, the preparation method typically involves intricate multistep polymerization or chemical modification. For example, the core–shell quantum dots MIPs were typically prepared by a time-consuming and complicated two steps, that is, the core–shell micelles were preprepared firstly and then differently fluorescent components were embedded into the core or shell [32,37]. This typical strategy is hard to control and not suitable for large-scale production to meet the increasing demands in various applications [31,38]. Specifically, the selective identification requirement of fluorescent MIPs for small organic molecules arises from aqueous bioanalysis systems [13,17,39,40,41]. Therefore, it is imperative to develop a facile preparation method for ratiometric fluorescent MIP sensors that exhibit exceptional performance in terms of direct, rapid, and sensitive detection capabilities.

Herein, we present a one-pot method for synthesizing a ratiometric fluorescent imprinted polymer nanoparticle sensor suitable for aqueous system. This strategy incorporates red fluorescent CdTe quantum dots (QDs) and green fluorescent nitrobenzodiazole (NBD) into an MIP nanosensor, enabling direct, reliable, sensitive, and selective quantitative analysis of small organic analytes in complex aqueous solutions. The selected target template analyte was 2,4-dichlorophenoxyacetic acid (2,4-D), which is widely used as an herbicide in crops and weeds despite its endocrine-disrupting activity [42,43,44,45]. By combing the hydrophilic macromolecular polyglyceryl methacrylate chain transfer agent (PGMMA-CTA) and hydrophobic cumyl dithiobenzoate (CDB), we successfully adjusted the reversible addition-fragmentation chain transfer radical precipitation polymerization (RAFTPP) to prepare a hydrophilic 2,4-D imprinted polymer with dual-fluorescence properties. The successful synthesis was confirmed through comprehensive characterization, including morphology analysis, particle size distribution measurement, chemical structure determination, surface hydrophilicity evaluation, balanced template binding assessment, and selectivity testing, along with fluorescence property examination. Furthermore, by introducing red QDs and green NBD as light sources for ratiometric fluorescence detection purposes, background noise was effectively eliminated while significantly enhancing detection accuracy.

## 2. Materials and Methods

### 2.1. Materials

The 4-vinylpyridine (4-VP, 96%, Alfa Aesar, Shanghai, China) was purified through vacuum distillation. 2,4-Dichlorophenoxyacetic acid (2,4-D, 98%) was obtained from Heowns Biochem Technologies LLC., Tianjin, China. Ethylene glycol dimethacrylate (EGDMA, Alfa Aesar, 98%) underwent washing with a solution of aqueous sodium hydroxide (10%) and water before being dried using anhydrous magnesium sulfate and subsequently distilled under vacuum conditions. 2,2′-Azobisisobutyronitrile (AIBN) obtained from the Chemical Plant of Nankai University was recrystallized from ethanol. Glyceryl monomethacrylate (GMMA) was synthesized by opening the epoxy ring of glycidyl methacrylate (GMA, 98%, Aladdin, Shanghai, China) using perchloric acid as described in previous work [46,47]. Cumyl dithiobenzoate (CDB) was prepared by synthesizing benzodithioic acid through the reaction between phenylmagnesium bromide and carbon disulfide followed by its subsequent reaction with *α*-methylstyrene according to a previously reported method [48]. Octaalkyldecyl-*p*-vinylbenzyl dimethylammonium chloride (OVDAC) was prepared involving reflux conditions for the reaction between *N*,*N*-dimethyl octadecylamine and 4-vinylbenzyl chloride in acetone. An aqueous solution of CdTe quantum dots functionalized with 3-mercaptopropionic acid was also prepared [49]. *p*-Hydroxybenzoic acid (POAc, 98%), 4-chlorophenoxyacetic acid (CPOAc, 98%), and 2,4-dichlorophenylacetic acid (DCPAc, 99%) were purchased from Shanghai Dibai Chemical Technology Co., Ltd. (Shanghai, China). Glucose (Howns Biochem Technologies Co., Ltd., Tianjin, China, 99%), l-glutamic acid (98%, Tianjin Sinos Aodepu Technology Co., Ltd., Tianjin China), and l-cysteine (99%, Tianjin Sinos Aodepu Technology Co., Ltd., Tianjin, China), along with other experimental reagents mentioned in this work, were used directly without any further treatment. All the real environmental water, including drinking water, lake water, urban runoff water, and paddy field water, was sampled from the surface water intakes in Tianjin, China. The structures of key reactants are shown in Scheme S1 in Supporting Information.

### 2.2. Synthesis

Synthesis of modified red cadmium telluride quantum dots (QDs)

To synthesize red cadmium telluride quantum dots (QDs), the method described in [49] was followed. In a 250 mL round-bottomed three-necked flask, cadmium chloride hydrate (41.34 g), 100 mL of purified water, and mercaptopropionic acid (45.2 μL) were combined. The pH was adjusted to 10.5~11 using a 1.0 mol/L sodium hydroxide aqueous solution. The mixture was vigorously stirred at room temperature for 5 min, followed by the addition of an aqueous solution of sodium tellurite (19.72 g) while continuing stirring for another 5 min. The pH was maintained, and sodium borohydride (160 mg) was added before sonication for 5 min. Refluxing at 120 °C in an oil bath resulted in the formation of red quantum dots with the desired wavelength (λ = 680 nm). Subsequently, OVDAC (120 mg) was introduced into a separate darkened single-necked round-bottomed flask containing 60 mL of red quantum dots and magnetically stirred for 2 h. Finally, chloroform (60 mL) was used for extraction and liquid separation; the organic phase was collected and subjected to three subsequent replacements with equal volumes of methanol. The intensity-averaged lifetime and quantum yield of QDs are 18.96 ns and ≥90%, respectively.

Synthesis of macromolecular RAFT chain transfer agent

A macromolecular chain transfer agent, polyglyceryl methacrylate (PGMMA-CTA, *M*_n_ = 8830 g/mol), with disulfide bonds was synthesized through the following steps: GMMA (13.48 mmol), AIBN (0.046 mmol), methanol (60 mL), and CDB (0.081 mmol) were successively added to a 100 mL single-necked round-bottomed flask and magnetically stirred at room temperature for 5 min until complete dissolution. The solution was then immersed in an ice-water bath and purged with argon for 30 min to remove air from the system, followed by placing the reaction flask in an oil bath at 60 °C and stirring for 48 h. After completion of the reaction, the mixture underwent three ether washes in an ice-water bath and was subsequently vacuum-dried at room temperature for 48 h, resulting in a pink powdery solid of 75%. The obtained product was characterized by NMR analysis, as shown in Figure S1.

Synthesis of NBD and QD-labeled 2,4-D-imprinted polymer (i.e., 2,4-D-MIP)/control polymer (2,4-D-CP) nanoparticles with surface-grafted PGMMA brushes

The synthesis of 2,4-D-imprinted polymer (2,4-D-MIP)/control polymer (2,4-D-CP) nanoparticles labeled with NBD and QDs and surface-grafted with polymethyl methacrylate (PGMMA) brushes was achieved through the RAFTPP, method employing a PGMMA-CTA RAFT agent. The synthesis procedure involved sequentially adding 4-VP (1.668 mmol), PGMMA-CTA (0.0684 mmol), a freshly prepared modified quantum dots solution (60 mL), NBD (1 mg), and a mixture of methanol and water (2:1 *v*/*v*, 90 mL) to a round-bottomed flask (250 mL). After stirring for 30 min at room temperature, a clear solution was obtained followed by the addition of EGDMA (5.02 mmol), AIBN (0.0566 mmol), and CDB (0.1104 mmol). The reaction mixture was purged with argon for 30 min before being sealed and immersed in a constant temperature oil bath at 60 °C for 24 h under magnetic stirring. The resulting polymer particles were collected by centrifugation, thoroughly washed with methanol until no template was detected in the supernatant after centrifugation, and freeze-dried under vacuum for 48 h to obtain brown QD- and NBD-labeled 2,4-D-MIP nanoparticles with surface-grafted PGMMA brushes (grafted QD, NBD-labeled 2,4-D-MIP, yield: 15%). Similarly, the corresponding dual-fluorescence-labeled nanoparticles of QD,NBD-2,4-D-CP with surface-grafted PGMMA brush (brown) were synthesized and purified following the same procedure but without adding any 2,4-D (yield: 16%).

### 2.3. Characterization

The chemical structures of samples were characterized using a Bruker TENSOR 27 FT-IR spectrometer (Bruker, Billerica, MA, USA). Each spectrum was obtained by averaging 64 scans, which were recorded at a resolution of 4 cm^−1^ within the range of 400 to 4000 cm^−1^. Scanning electron microscope (SEM) analysis was conducted using an FEI Nano SEM 450 scanning electron microscope with an accelerating voltage of 600 V. The preparation process of the sample film for static contact angle test was as follows: the sample was evenly dispersed in dimethyl formamide by ultrasound (10 mg/mL), and then coated on a clean slide, then the solvent was volatilized at room temperature, and then dried in a vacuum oven at 40 °C for 48 h. Finally, the static contact angle was measured on a Static water contact angle meter (SDC-2000) (INDRA, Madrid, Spain) at 25 °C, and each sample was tested three times for an average value. High-performance liquid chromatography (HPLC, Agilent 1100 Series, Agilent, Santa Clara, CA, USA) was employed to evaluate the equilibrium and selective binding properties of modified and unmodified dual-fluorescent MIP in both organic (methanol/water = 4:1, *v*:*v*) and pure water phases after incubation with the analyte. Fluorescence emission measurements of grafted (and ungrafted) dual-fluorescent 2,4-D-MIP/CP particle solutions mixed with their respective analytes were conducted using a Tokyo Hitachi F-4600 (Hitachi, Tokyo, Japan) fluorescence spectrometer at an excitation wavelength of 420 nm and voltage of 600 V. Furthermore, light-sensing properties of grafted (and ungrafted) dual-fluorescent 2,4-D-MIP/CP particles were examined through spectroscopy by studying the width-matched excitation and emission slits set at 10 nanometers.

## 3. Results and Discussion

### 3.1. Synthesis of Hydrophilic 2,4-D-MIP/2,4-D-CP Colloidal Nanoparticles with Dual-Fluorescent Labels

**Figure 1:**
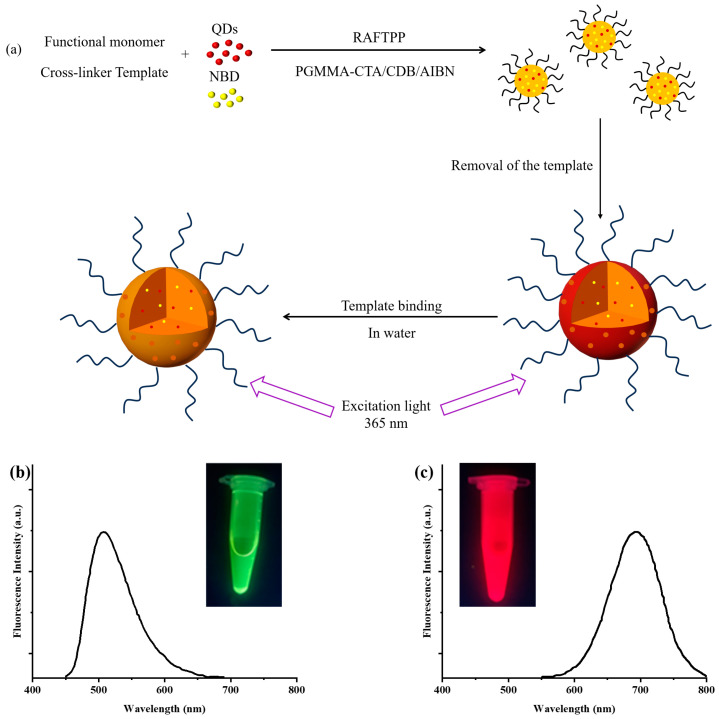
One-step synthetic route for hydrophilic quantum dots labeled fluorescent MIP nanoparticles and their schematic diagrams (**a**); NBD (**b**) and red quantum dots (**c**) fluorescence spectra and optical images under UV light.

One-step synthesis of hydrophilic polymethyl methacrylate brush-grafted NBD and QD-labeled 2,4-D imprinted polymer nanoparticles (grafted NBD, QD-labeled 2,4-D-MIP in Table 1, entry 1) was achieved through the RAFTPP under the mediation of a hydrophilic macromolecular PGMMA-CTA (Figure 1). QDs and NBD were utilized as templates while 4-VP, EGDMA, CDB, and AIBN acted as functional monomers, cross-linking agents, RAFT chain transfer agents, and initiators, respectively. The well-defined hydrophilic macromonomer PGMMA-CTA serves as both a co-RAFT agent and a steric stabilizer during the RAFTPP process. This effectively facilitates the incorporation of hydrophilic polymer brushes onto the resulting dual-fluorescent-labeled 2,4-D-molecularly imprinted polymer particles. Furthermore, CDB was employed as a co-RAFT reagent to precisely control the morphology, size, and polydispersity of the resulting molecularly imprinted polymer particles. The corresponding control polymer of grafted NBD, QD-labeled 2,4-D-CP was synthesized following identical experimental conditions (Table 1, entry 2), with the exception of omitting the template molecule (2,4-D). Moreover, a double-fluorescent-labeled ungrafted NBD, QD-labeled MIP/CP (serving as a control for grafted NBD, QD-labeled MIP) was also synthesized (Table 1, entries 3 and 4).

**Figure 2:**
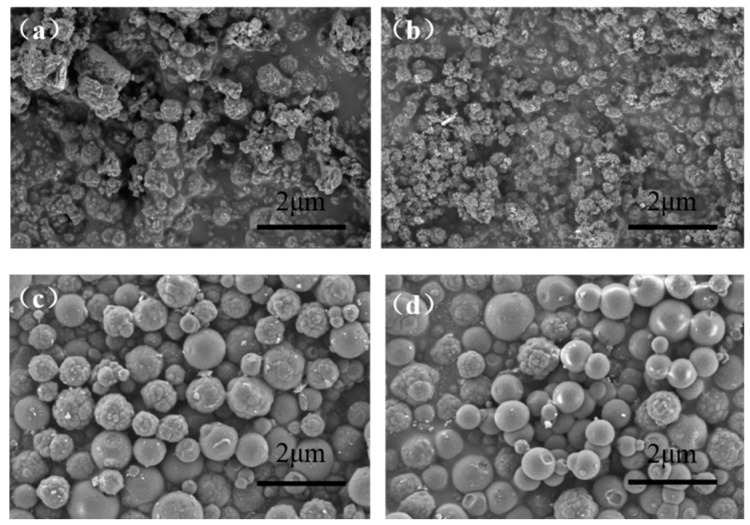
SEM images of dual-fluorescent polymers of grafted QD,NBD-labeled 2,4-D-MIP (**a**), grafted QD,NBD-labeled 2,4-D-CP (**b**), ungrafted QD,NBD-labeled 2,4-D-MIP (**c**), and ungrafted QD, NBD-labeled 2,4-D-CP (**d**).

The hydrophilic 2,4-D-MIP and 2,4-D-CP labeled with NBD and QD were confirmed to be colloidal nanoparticles based on DLS analysis, demonstrating particle sizes of 215 nm and 232 nm, respectively. Conversely, the absence of a macromolecular stabilizer in PGMMMA-CTA resulted in larger particle sizes for ungrafted NBD and QD-labeled 2,4-D-MIP/2,4-D-CP at 966 nm and 865 nm, respectively. This observation is also supported by the scanning electron micrographs (SEMs) presented in Figure 2. The MIP/CP without hydrophilic molecular brushes displayed a smoother surface with larger size, whereas the presence of hydrophilic molecular brushes on the polymer surface led to smaller size due to the adjustment effect of macromolecular chain transfer agent and a rougher surface texture (Figure 2a,b). Furthermore, prominent peaks corresponding to hydroxyl groups (at approximately 3450 cm^−1^ for OH stretch and at 1427 cm^−1^ for OH in-plane bending) observed in the infrared spectra further validated the existence of PGMMA brushes on grafted NBD and QD-labeled 2,4-D-MIP/2,4-D-CP nanoparticles (Figure S2).

**Figure 3:**
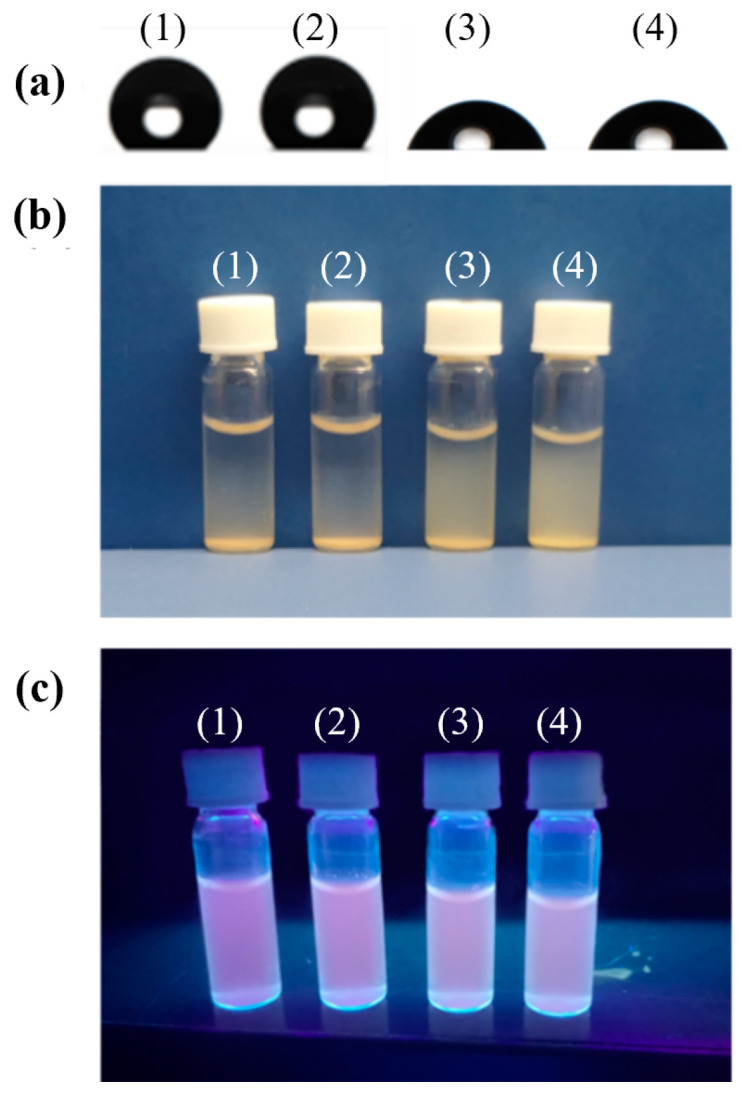
Profiles of a water drop on the films of the ungrafted/grafted dual-fluorescent 2,4-D-MIPs/2,4-D-CPs (**a**), their dispersion photograph in pure water (1 mg/mL) at 25 °C after settling down for 5 h (under daylight irradiation) (**b**), and photograph of their homogeneously dispersed samples in water (1 mg/mL) under 365 nm UV light irradiation (**c**). The samples in each figure are arranged from left to right as ungrafted dual-fluorescent 2,4-D-MIP (1)/2,4-D-CP (2) and grafted dual-fluorescent 2,4-D-MIP (3)/2,4-D-CP (4).

Additionally, the utilization of macromolecular chain transfer agent PGMMA-CTA in the preparation process enhances colloidal particles stability. Figure 3 demonstrates that compared to ungrafted NBD, QD-labeled 2,4-D-MIP/2,4-D-CP nanoparticles, the grafted exhibit reduced static membrane contact angles (Figure 3a), thereby enhancing their dispersion stability in pure water (Figure 3b and Figure S3). Moreover, the fluorescence spectrum reveals an intense emission peak and strong fluorescence upon irradiation with 365 nm ultraviolet light (Figure 3c), providing further evidence for successful binding of NBD and CdTe quantum dots to these grafted molecularly imprinted polymer particles. Consequently, incorporation of PGMMA-CTA facilitates the development of small and stable colloidal MIP and CP nanoparticles, which is highly advantageous for selective detection of trace amounts of pesticide 2,4-D as nanosensors [50,51].

### 3.2. Equilibrium Adsorption Performance of Grafted and Ungrafted Dual-Fluorescently Labeled Molecularly Imprinted Polymers

**Figure 4:**
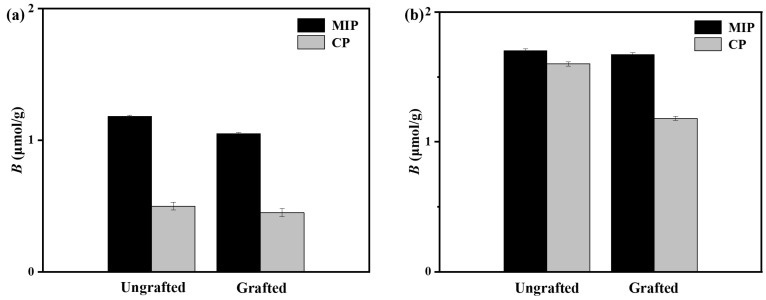
Equilibrium adsorption of ungrafted and grafted nanosensors in methanol/water (4/1, *v*/*v*) (**a**) and pure water (**b**).

As shown in Figure 4, the equilibrium adsorption test demonstrates that hydrophilic grafted NBD,QD-labeled 2,4-D-MIP exhibits a higher template binding capacity compared to hydrophilic grafted NBD,QD-labeled 2,4-D-CP in both the organic phase (methanol/water = 4:1, *v*/*v*) and pure water. This observation confirms the presence of specific binding sites and a successful molecular imprinting process. Importantly, the surface modification with a hydrophilic PGMMA brush significantly enhances the surface hydrophilicity of grafted QD,NBD-labeled 2,4-D-MIP/2,4-D-CP while maintaining their selective adsorption towards 2,4-D. Furthermore, we evaluated the competitive binding ability of ungrafted and grafted NBD,QD-labeled 2,4-D-MIP/2,4-D-CP towards 2,4-D and its structural analogue of *p*-hydroxybenzoic acid (POAc) (Figure S4). Their equilibrium binding capacity (*B*) is the key parameter for estimating the selective ability [32]. These results demonstrated that grafted NBD,QD-labeled 2,4-D-MIPs exhibit remarkable selectivity in both organic solvents and aqueous solutions, whereas ungrafted NBD,QD-labeled 2,4-D-MIPs only show selectivity to 2,4-D in organic phase. It can be concluded that only brush-modified nanoparticles of grafted NBD,QD labeled-2,4-D-MIPs with high surface hydrophilicity can be employed as chemical nanosensors for detecting specific fluorescent antibodies in water samples.

### 3.3. Optical Sensing Properties of Grafted NBD,QD-Labeled 2,4-D-MIP/2,4-D-CP Nanoparticles in Aqueous System

In the equilibrium adsorption experiment, it was confirmed that only the NBD,QD-labeled 2,4-D-MIP grafted with hydrophilic molecular brushes exhibited specific recognition ability towards the template in aqueous system. This phenomenon can be attributed to the significant enhancement of hydrophilicity on the polymer surface achieved through surface modification. Consequently, our subsequent investigations focused solely on conducting an in-depth characterization of the fluorescence properties of double fluorescent imprinted polymer nanoparticles grafted with hydrophilic molecular brushes.

**Figure 5:**
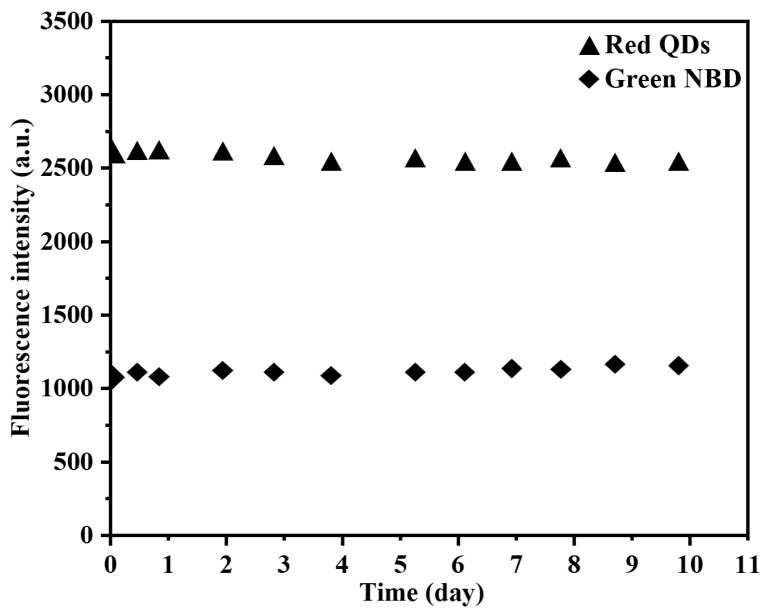
The fluorescence intensity of red QDs (triangle) and green NBD (rhombus) in hydrophilic ratio fluorescent MIP microspheres over time. The concentration of MIP microspheres is 0.50 mg/mL in water.

**Figure 6:**
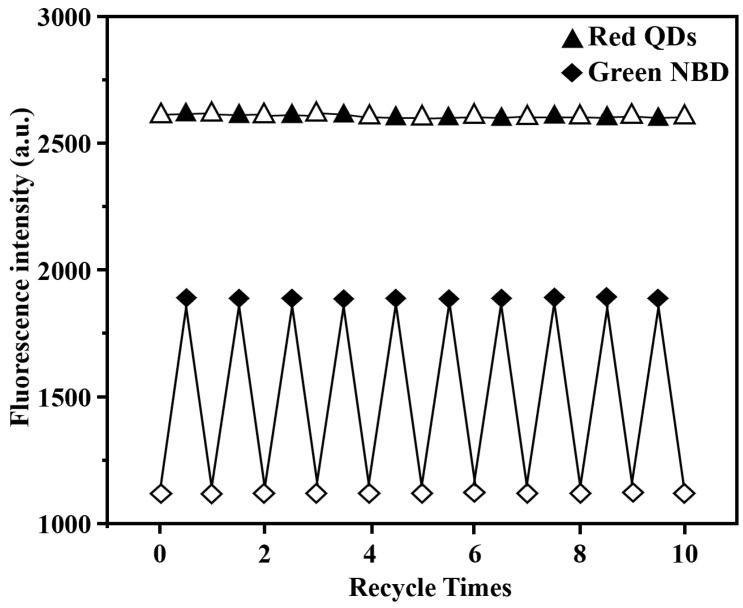
Fluorescence intensity changes of the grafted dual-fluorescent 2,4-D-MIP (0.50 mg/mL) during its 10 regeneration cycles. Red QDs (triangle) and green NBD (rhombus) undergo desorption (empty) and adsorption (filled) of 2,4-D (20 μM) in the aqueous solution.

The fluorescence stability and reusability of the dual-fluorescent polymer were characterized in an aqueous system. After a storage period of 10 days, negligible changes in the intensity of both NBD and red QDs fluorescence peaks were observed for the dual-fluorescent polymer, indicating its excellent fluorescence stability (Figure 5). Furthermore, during the adsorption–desorption cycle test conducted over 10 cycles, NBD fluorescence intensity exhibited consistent cyclic behavior while the red quantum dots’ fluorescence intensity remained essentially unchanged (Figure 6). These results provide evidence for the exceptional reusability performance of the fluorescence nanosensor.

The grafted dual-fluorescence 2,4-D-MIP/2,4-D-CP was incubated with an aqueous solution of 2,4-D to investigate the temporal changes in their fluorescence and elucidate their binding kinetics. Upon combination with 2,4-D in the aqueous solution, the grafted dual-fluorescence 2,4-D-MIP exhibited a remarkable fluorescence enhancement at 500 nm (induced by the NBD fluorophore), reaching its maximum intensity within approximately 30 min (Figure S5). The observed fluorescence amplification effect in this study exceeded the levels reported in previous studies for the corresponding 2,4-D-CP [52]. This study demonstrates that molecularly imprinted polymers have a rapid light-sensing process and significant practical applications. Furthermore, negligible alterations in fluorescence were observed at 680 nm for both the grafted dual-fluorescence 2,4-D-MIP and 2,4-D-CP systems when paired with red CdTe quantum dots, thus confirming their inert nature through fluorescence titration experiments.

**Figure 7:**
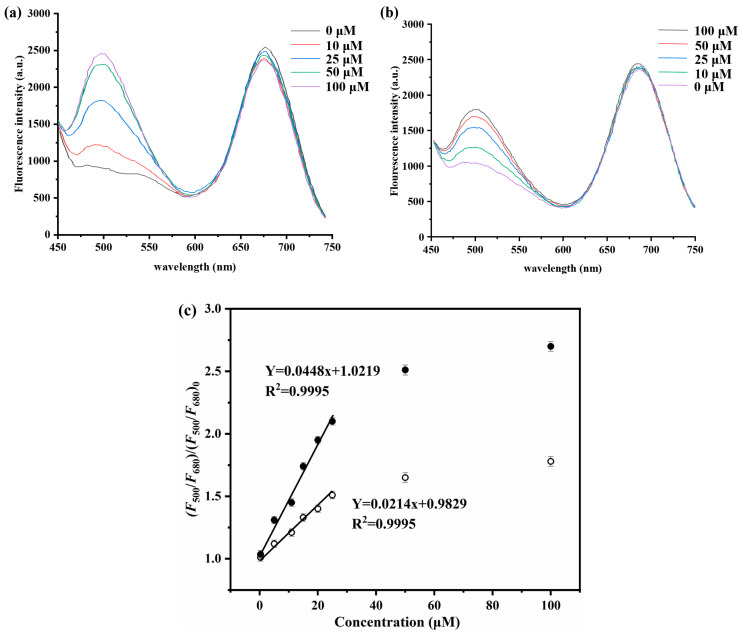
Fluorescence spectra of grafted QDs, NBD-labeled 2,4-D-MIP (**a**), and grafted QDs, NBD-labeled 2,4-D-CP (**b**) (0.50 mg/mL) upon their incubation with different concentrations of 2,4-D in pure water; (**c**) the fluorescence enhancement of grafted dual-fluorescent 2,4-D-MIP (filled symbol)/2,4-D-CP (open symbol) depending on 2,4-D concentration (derived from (**a**,**b**)).

The fluorescence titration experiment was conducted on the double-fluorescent molecularly imprinted polymer grafted in the aqueous solution, and the resulting data were collected and are presented in Figure 7 and Table S1. The fluorescence intensity of the green NBD fluorophore introduced at 500 nm was found to increase proportionally with the concentration of 2,4-D, while the fluorescence intensity of the QD introduced at 680 nm remained relatively constant regardless of changes in 2,4-D concentration. Therefore, there is an observed increase in the ratio of fluorescence intensity at 500 nm to that at 680 nm (i.e., *F*_500_/*F*_680_) as a function of increasing concentrations of 2,4-D. This is a typical characteristic of turn-on mechanism, that is, once the analyte of 2,4-D binds onto MIP, an obvious fluorescence enhancement at 500 nm can be recognized, whereas that of their incorporated QDs at 680 nm remained almost constant following the change of the 2,4-D concentration. Therefore, the ratio of *F*_500_/*F*_680_ increased with increasing 2,4-D concentration. The fluorescence amplification may be achieved by both the specific and stronger interaction between the imprinted cavities and 2,4-D and the nonspecific binding, thus leading to a larger fluorescence change in the binding processes for the grafted dual-fluorescent 2,4-D-MIP [53]. Based on the equation (*F*_500_/F_680_)/(*F*_500_/*F*_680_)_0_ = KC + 1 [32], the green NBD and red CdTe quantum dots (*F*_500_/*F*_680_)/(*F*_500_/*F*_680_)_0_ are established for the 2,4-D concentration (in the linear graph of the fluorescence intensity ratio within the concentration range of 0–25 μM). 2,4-D is used as the calibration curve of grafted dual-fluorescence 2,4D-MIP, where (*F*_500_/*F*_680_) and (*F*_500_/*F*_680_)_0_ are the concentration ratios of fluorescent green NBD and red cadmium telluride quantum dots in the presence and absence of 2,4-D, K is a constant, and C is 2,4-D concentration. Thus, the linear response range of ratiometric fluorescence MIP to the template molecule of 2,4-D ranges from 0 to 25 μM. The limit of detection (LOD) of the grafted dual-fluorescence 2,4-D-MIP optical sensor was determined to be 0.13 μM (in pure water), and the standard deviation (20 times) of the blank measurement value. This LOD reached a high-level sensitivity of double fluorescent MIP, while exceeded most of the existing detection methods ranging from 0.02 μM to 20.00 μM, thereby indicating the suitability of this method for trace detection of 2,4-D in municipal wastewater (approximately 0.05–0.50 μM) [1,54,55]. Three times (3σ) divided by the slope of the experimentally determined calibration chart, which is lower than the drinking water (i.e., 0.32 μM or 70 μgL) prescribed by the World Health Organization (WHO) and the U.S. Food and Drug Administration (U.S.) [56]. By using the equation *K*_MIP_/*K*_CP_ (the slope of the calibration curve of MIP and its CP in Figure 7c for *K*_MIP_ and *K*_CP_, respectively), the imprinting factor of the grafted dual-fluorescence 2,4-D-MIP was derived as 2.1 in pure water. These results once again confirm the presence of the imprinting binding site in the grafted dual-fluorescence 2,4-D-MIP and its high 2,4-D recognition ability in the aqueous system.

Additionally, the results depicted in Figure 7a–c demonstrate that, under identical conditions, the grafted dual-fluorescence 2,4-D-MIP exhibits a significantly higher fluorescence enhancement at 500 nm compared to its CP counterpart. This observation suggests that the magnitude of fluorescence enhancement is influenced by both the MIP/CP particles and the binding affinity of the herbicide template. Notably, weak and nonspecific binding interactions dominate between the grafted dual-fluorescence 2,4-D-CP and 2,4-D molecules. In contrast, for the grafted dual-fluorescence 2,4-D-MIP system, fluorescence enhancement arises from specific and strong interactions occurring within the imprinting cavity between 2,4-D and nonspecifically bound counterparts. Consequently, a substantial change in fluorescence intensity is observed during the binding process of grafted dual-fluorescence 2,4-D-MIP. It should be noted that when combined with aqueous solutions containing 2,4-D, the ungrafted dual-fluorescence 2,4-D-MIP shows negligible changes in fluorescence intensity at wavelengths of interest (500 nm).

**Figure 8:**
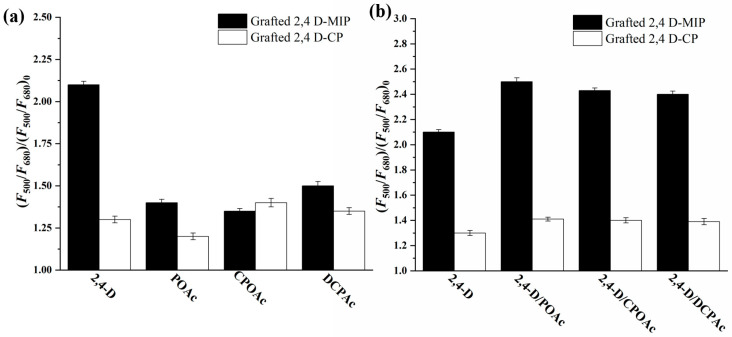
Fluorescence enhancement of the grafted dual-fluorescent 2,4-D-MIP (filled column)/2,4-D-CP (open column) (0.50 mg/mL) after their incubation with a solution of 2,4-D (50 μM) in the presence of 1 (**a**) and 10 (**b**) equivalents of POAc, CPOAc, or DCPAc in aqueous solution.

The selective light-sensing ability of grafted dual-fluorescence 2,4-D-MIP/CP was evaluated by comparing their fluorescence enhancement (i.e., (*F*_500_/*F*_680_)/(*F*_500_/*F*_680_)_0_) upon exposure to 2,4-D and its structurally related analogues, including POAc, 4-chlorophenoxyacetic acid (CPOAc) and 2,4-dichlorophenylacetic acid (DCPAc), in aqueous solution. As depicted in Figure 8a, the grafted dual-fluorescence 2,4-D-MIP exhibited significantly higher fluorescence enhancement compared to its analogues. Moreover, while the fluorescence enhancement of the grafted dual-fluorescence 2,4-D-MIP towards 2,4-D was considerably greater than that of its corresponding counterpart—2,4-D-CP, both showed nearly identical fluorescence enhancements towards the analogues of 2,4-D. The comparison suggests the presence of imprinted binding in the grafted 2,4-D MIP, and the slight fluorescence enhancement observed upon exposure to POAc, CPOAc, and DCPAc is primarily attributed to nonspecific bindings.

The selectivity of grafted dual-fluorescence 2,4-D-MIP/CP in light sensing was further evaluated by examining their fluorescence enhancement to 2,4-D in complex aqueous solutions containing the abovementioned structural analogues of POAc, CPOAc, and DCPAc. Further results revealed that even when there were 1 to 10 equivalents of analogues present in the 2,4-D solution (Figure 8b), the interference with the light-sensing capability of grafted dual-fluorescent 2,4-D-MIP towards 2,4-D was negligible. These results unequivocally demonstrate that the grafted double fluorescent 2,4-D-MIP holds great promise as a highly selective “on” ratio fluorescent light-sensing material for direct and specific detection of 2,4-D. The validity of this finding was further confirmed in a complex mixed solution containing glucose, l-glutamic acid, and l-cysteine at concentrations ten times higher than that of 2,4-D (Figure S6). This simplified simulation of a biological system serves to strengthen the robustness and applicability of our results. Additionally, we demonstrated the direct and selective optosensing of 2,4-D in various real environmental water samples, including drinking water, lake water, urban runoff water, and paddy field water (Table S2). Urban runoff water and paddy field water were found to contain trace amounts of 2,4-D with concentrations of 0.15 ± 0.01 μM and 0.95 ± 0.05 μM, respectively, indicating the use of herbicides in these areas. No detectable levels of 2,4-D were observed in the drinking water and lake water samples, although spiked samples with different amounts of 2,4-D were used for recovery tests to ensure highly accurate detection concentrations. Excellent recoveries ranging from 99.2% to 100.8% were achieved for all aqueous solutions tested, with standard deviations (RSDs) ranging from 1.5% to 3.3%. Thus, this work provides robust evidence supporting the exceptional efficacy of our dual-fluorescent-labeled hydrophilic molecularly imprinted polymer as an advanced chemical sensor capable of direct, selective, and precise detection of small molecules in complex aqueous environments.

## 4. Conclusions

In conclusion, the colloidal ratiometric fluorescent MIP nanoparticles were successfully synthesized using the facile one-pot RAFTPP method. The synergistic collaboration between chain transfer agents CDB and PGMMA-CTA resulted in the formation of uniform-sized MIP micelles with enhanced hydrophilicity, thereby facilitating the fabrication of highly sensitive and selective optical chemical nanosensors for 2,4-D by incorporating dual fluorophores of NBD and QD. Comparative analysis against control samples lacking imprinted monomers or PGMMA-CTA revealed the exceptional recognition ability of the grafted dual-fluorescence 2,4-D-MIP nanosensors towards this herbicide in aqueous systems due to the presence of imprinting binding sites. Moreover, these advanced dual-fluorescence molecularly imprinted nanoparticles exhibited outstanding light-sensing capabilities for fluorescence detection in complex aqueous samples while effectively eliminating noise from analogues. Consequently, the grafted dual-fluorescence 2,4-D-MIP optical sensors achieved a remarkable detection limit of 0.13 μM for 2,4-D in pure water. These findings hold significant promise for their application as optical chemical sensors across diverse practical domains, such as clinical diagnosis and food analysis.

## Figures and Tables

**Table 1:** Characteristic parameters of hydrophilic double fluorescent-labeled 2,4-D molecularly imprinted polymers.

Entry	MIP/CP *^a^*	Yield/(%)	*D*_n_/(nm) *^b^*	Contact angle *^c^*/(°)
1	Grafted QD, NBD-labeled 2,4-D-MIP	15	215	70.3
2	Grafted QD, NBD-labeled 2,4-D-CP	14	232	72.1
3	Ungrafted QD, NBD-labeled 2,4-D-MIP	25	966	120.2
4	Ungrafted QD, NBD-labeled 2,4-D-CP	24	865	120.7

*^a^* QD and NBD labeled polymer with a hydrophilic PGMMA brush on the surface (grafted QD, NBD labeled 2,4-D-MIP/CP, entries 1 and 2) by simultaneous addition of CDB and PGMMA-CTA; hydrophilic brush-free quantum dot labeled 2,4-D binding polymer (ungrafted NBD, rQD labeled 2,4-D-MIP/CP, entries 3 and 4) by exclusive addition of CDB; *^b^ D*_n_ is the quantum dot label with or without hydrophilic brush on the surface measured by DLS. *^c^* Static water contact angle measured by testing polymer membranes.

## Data Availability

All relevant data are within the manuscript and its Additional Supplementary Materials.

## Supplementary Materials

The following supporting information can be downloaded at: https://www.mdpi.com/article/10.3390/s24155039/s1, Scheme S1: The structures of key reactants in this study; Figure S1: ^1^H NMR spectrum of PGMMA-CTA in DMSO-*d*_6_; Figure S2: FT-IR spectra of 2,4-D MIP labeled with quantum dots: grafted QD,NBD-labeled 2,4-D-MIP (a), grafted QD,NBD-labeled 2,4-D-CP (b), ungrafted QD,NBD-labeled 2,4-D-MIP (c) and ungrafted QD,NBD-labeled 2,4-D-CP (d); Figure S3: Photographs of the ultrasonically dispersed aqueous mixtures (1.0 mg/mL) after their being settled down for 0 h (a), 1 h (b), 2 h (c), 3 h (d), 7 h (e) and 12 h (f), respectively. The samples in each figure are arranged from left to right as ungrafted dual-fluorescent 2,4-D-MIP/2,4-D-CP and grafted dual-fluorescent 2,4-D-MIP/2,4-D-CP; Figure S4: Selective adsorption results of 2,4-D and POAc mixed solution of polymer in methanol/water (4/1, *v*/*v*) (a) and pure water (b); Figure S5: The fluorescence intensity of hydrophilic ratio fluorescent MIP/CP microspheres in 2,4-D aqueous solution over time. (a) Grafted QD, NBD-labeled 2,4-D-MIP, (b) Grafted QD, NBD-labeled 2,4-D-CP. (c) Fluorescence dynamics of hydrophilic ratio fluorescent MIP/CP microspheres; Figure S6: Fluorescence enhancement of the grafted dual fluorescent (filled column)/2,4-D-CP (open column) after their incubation with a solution of 2,4-D in the presence of 1 (a), and 10 (b) equivalents of mixed Glucose, l-Cysteine, and l-Glutamic acid in aqueous solution at 25 °C for 30 min; Table S1: Calculation results of imprinted polymer’s IPB values; Table S2 The direct quantification of 2,4-D by grafted 2,4-D MIP sensors in different real environmental aqueous solution (drinking water, lake water, urban runoff water and paddy field water) with different amounts of 2,4-D.
