# Supplementary material for: One-Pot Preparation of Ratiometric Fluorescent Molecularly Imprinted Polymer Nanosensor for Sensitive and Selective Detection of 2,4-Dichlorophenoxyacetic Acid
Source: Sensors (Basel). 2024 Aug 3;24(15):5039. doi: 10.3390/s24155039 (PMC11315029; doi:10.3390/s24155039)
Supplement: Supplementary file 1 [file sensors-24-05039-s001.zip › sensors-3118215-Supplementary Information.pdf]

**Supporting information for**

# One-Pot Preparation of Ratiometric Fluorescent Molecularly Imprinted Polymer Nanosensor for Sensitive and Selective Detection of 2,4-Dichlorophenoxyacetic Acid

Yuhong Cui [1], Xintai Li [1], Xianhong Wang [2], Yingchun Liu [3], Xiuli Hu [1], Shengli Chen [1,*] and Xiongwei Qu [1,*]

1 Hebei Key Laboratory of Functional Polymers, School of Chemical Engineering and Science, Hebei University of Technology, Tianjin 300401, China

2 Tianjin Key Laboratory of New Materials and Systems for HVAC Plumbing, Tianjin 300400, China

3 Jinghua Plastics Co., Ltd., Langfang 065800, China

* Correspondence: shenglichen@hebut.edu.cn (S.C.); xwqu@hebut.edu.cn (X.Q.)

## 1. Materials and Samples

### 1.1 Materials

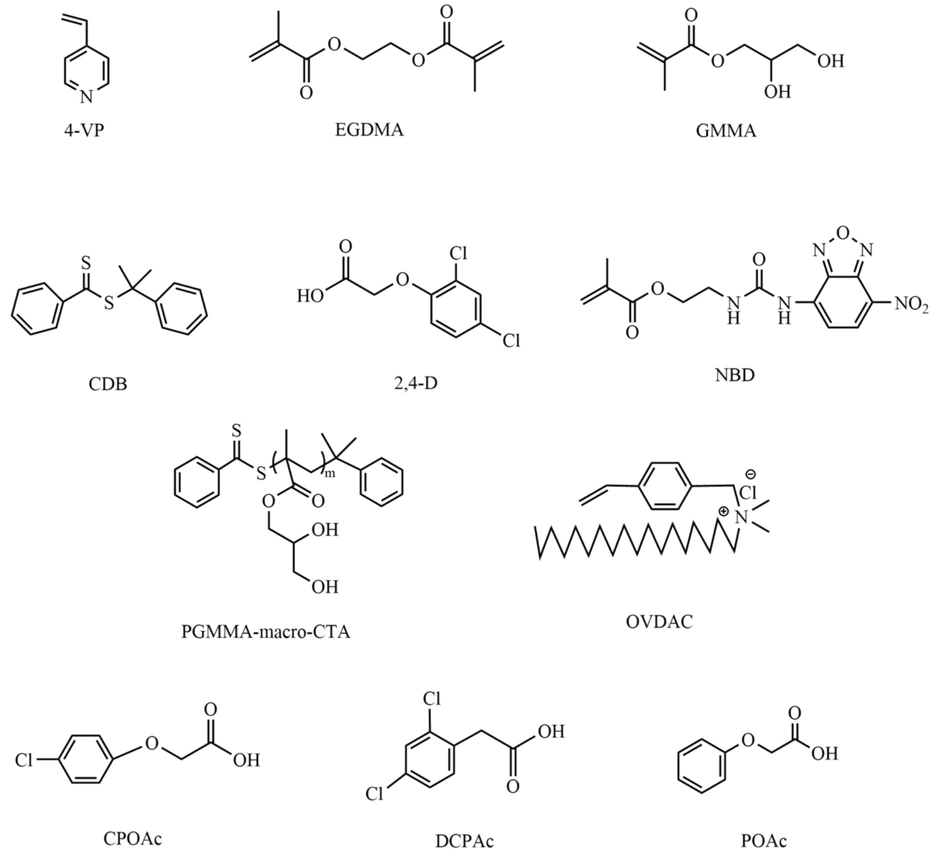


**Scheme S1.** The structures of key reactants in this study.

### 1.2 Abbreviations

| | | | |
|---|---|---|---|
| 4-VP | 4-vinylpyridine | MIP | molecular imprinted polymer |
| AIBN | 2,2'-azobisisobutyronitrile | 2,4-D | 2,4-dichlorophenoxyacetic acid |
| EGDMA | ethylene glycol dimethacrylate | PGMMA-CTA | polyglyceryl methacrylate chain transfer agent |
| GMA | glycidyl methacrylate | NBD | nitrobenzodiazole |
| GMMA | glyceryl monomethacrylate | QD | red cadmium telluride quantum dots |
| GMA | glycidyl methacrylate | 2,4-D-MIP | 2,4-D-imprinted polymer, adding 2,4-D as molecular template |
| CDB | cumyl dithiobenzoate | 2,4-D-CP | 2,4-D-control polymer, without adding 2,4-D as molecular template |
| OVDAC | octaalkyldecyl-p-vinylbenzyl dimethylammonium chloride | Grafted QD, NBD-labeled | 2,4-D-MIP nanoparticles with PGMMA brushs |

| | | | |
|---|---|---|---|
| | | 2,4-D-MIP | |
| POAc | *p*-hydroxybenzoic acid | | |
| CPOAc | 4-chlorophenoxyacetic acid | Grafted QD, NBD-labeled 2,4-D-CP | 2,4-D-CP nanoparticles with PGMMA brush |
| DCPAc | 2,4-dichlorophenylacetic acid | Ungrafted QD, NBD-labeled 2,4-D-MIP | 2,4-D-MIP nanoparticles without PGMMA brush |
| RAFTPP | reversible addition fragmentation chain transfer precipitation polymerization | Ungrafted QD, NBD-labeled 2,4-D-CP | 2,4-D-CP nanoparticles with PGMMA brush |

## 2. Characterization and Discussion

### 2.1 Calculation method for the number-average molecular weight of PGMMA-CTA

The number-average molecular weight ($M_n$) of polymer was determined to be 8830 g/mol using the following equation:

$$M_n = 2 \times (S_{d+c+e} / S_j) \times M_{GMMA} + M_{CDB}$$

$S_{d+c+e}$ represents the integrated area of peak 3.330-4.341 ppm, $S_j$ represents the integrated area of peak j (7-8 ppm), $M_{GMMA}$ represents the molecular weight of GMMA, and $M_{CDB}$ represents the molecular weight of CDB.

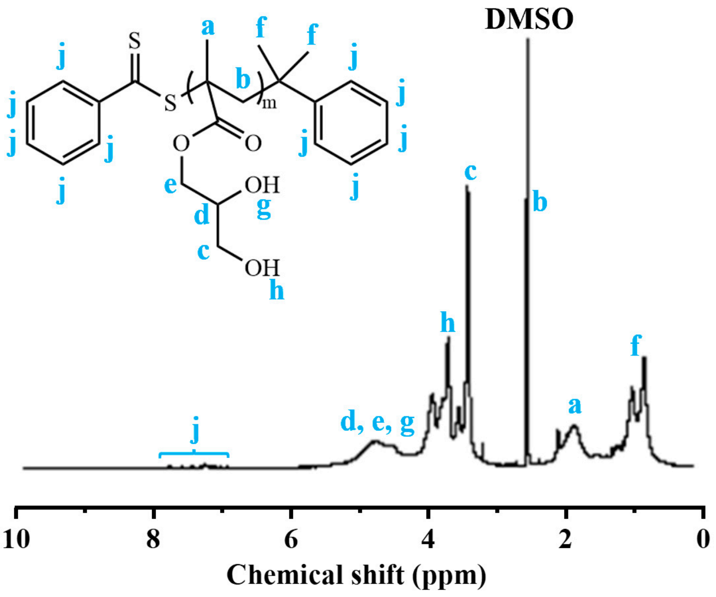


**Figure S1.** $^1$H NMR spectrum of PGMMA-CTA in DMSO-$d_6$.

## 2.2 FT-IR characterization

The FT-IR spectra of polymers with and without hydrophilic macromolecular brushes on the surface are shown in Figure S2. Figures S2a and S2b represent the infrared spectra of ungrafted QD, NBD-labeled 2,4-D-MIP, Ungrafted QD, NBD-labeled 2,4-D-CP without a macromolecular chain transfer agent. Figures S2c and S2d show the infrared spectra of grafted QD, NBD-labeled 2,4-D-MIP, grafted QD, NBD-labeled 2,4-D-CP with hydrophilic molecular brush on the surface. It can be observed from the figure that both samples exhibit similar chemical composition: a C=O stretching peak at 1730 $cm^{-1}$; a C-O-C stretching peak at 1155 $cm^{-1}$; a C=N stretching peak of the pyridine ring at 1156 $cm^{-1}$; and a C=C peak of the pyridine ring at 1456 $cm^{-1}$. Additionally, there is a prominent hydroxyl stretching vibration peak at 3450 $cm^{-1}$. The contact angles presented in Table 1 for polymers with hydrophilic brushes are significantly smaller compared to those without hydrophilic brushes. These findings indicate successful grafting of PGMMA polymer brushes onto the particle surfaces. It is worth noting that no characteristic peak corresponding to NBD is observed in the spectrum due to its low content (only comprising 5% of total content) within the polymer and limited sensitivity of our instrument.

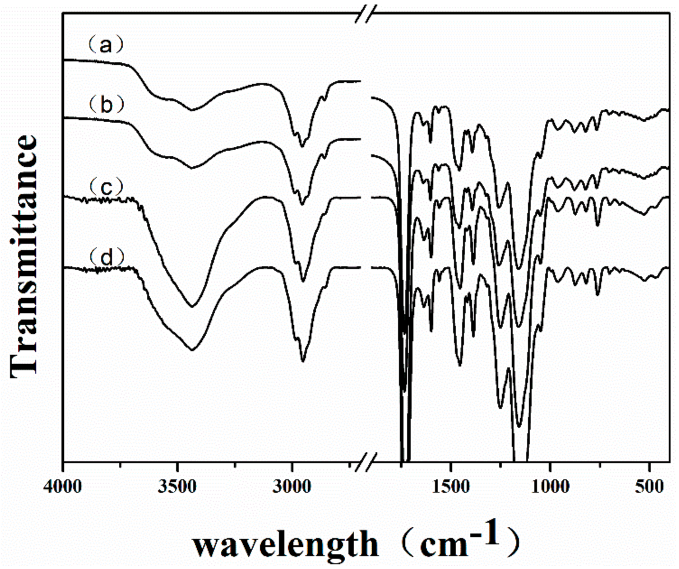


**Figure S2**. FT-IR spectra of 2,4-D MIP labeled with quantum dots: grafted QD,NBD-labeled 2,4-D-MIP (a), grafted QD,NBD-labeled 2,4-D-CP (b), ungrafted QD,NBD-labeled 2,4-D-MIP (c) and ungrafted QD,NBD-labeled 2,4-D-CP (d).

## 2.3 Suspension performance of ratio fluorescent labeled hydrophilic MIP microspheres in pure water

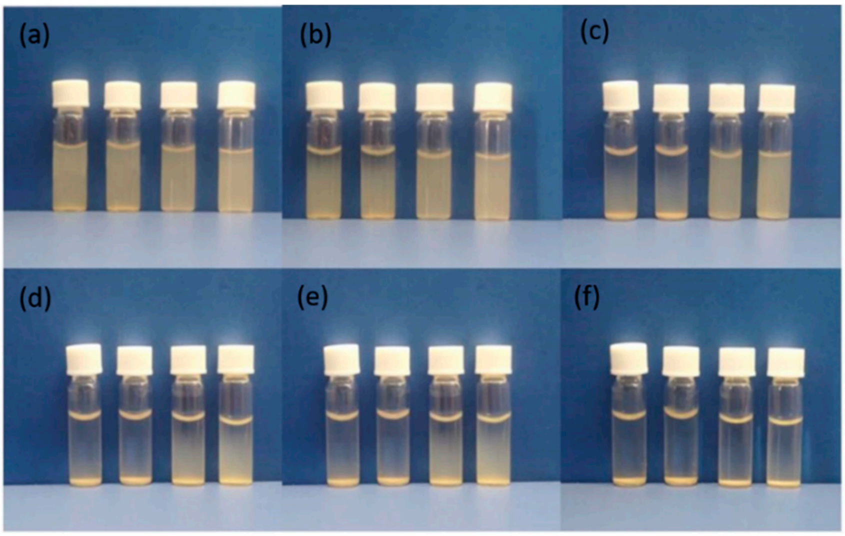


**Figure S3.** Photographs of the ultrasonically dispersed aqueous mixtures (1.0 mg/mL) after their being settled down for 0 h (a), 1 h (b), 2 h (c), 3 h (d), 7 h (e) and 12 h (f), respectively. The samples in each figure are arranged from left to right as ungrafted dual-fluorescent 2,4-D-MIP/2,4-D-CP and grafted dual-fluorescent 2,4-D-MIP/2,4-D-CP.

In generally, the grafting hydrophilic macromolecular brushes onto the surface of hydrophobic polymers can significantly enhance their hydrophilicity. Consequently, the polymer was formulated into a 1 mg/mL suspension in deionized water, subjected to ultrasonic dispersion for 10 minutes, and subsequently allowed to stand undisturbed at room temperature to observe its inherent aggregation and sedimentation behaviors in aqueous media, as illustrated in Figure S3.

## 2.4 Equilibrium binding and competitive binding experiments with the ungrafted and grafted dual fluorescent 2,4-D-MIPs/CPs in different media.

The adsorption equilibrium performance of MIP/CP in the organic phase (methanol/water=4/1, v/v) was evaluated as follows: 6 mg of Grafted QD, NBD-labeled 2,4-D-MIP and Grafted QD,NBD-labeled 2,4-D-CP microspheres were placed in a 2 mL plastic centrifuge tube along with ungrafted QD,NBD-labeled 2,4-D-MIP and ungrafted QD,NBD-labeled 2,4-D-CP microspheres. Subsequently, a solution containing a concentration of 0.02 mM of 2,4-D (1 mL) was added and incubated at a constant temperature (25 °C) for 8 hours. After centrifugation, the supernatant was collected and analyzed using HPLC with methanol-water

mobile phase (volume ratio:80/19.5/0.5), acetic acid mixed solution as eluent at a detection wavelength of 284 nm. Each sample was measured three times to obtain average values for calculating the adsorption amount B (μmol/g) of the template molecule - fluorescent molecularly imprinted polymer - by determining changes in peak area corresponding to the template molecule in solution. The adsorption equilibrium performance of MIP/CP in deionized water followed similar experimental conditions as those employed in the organic phase; however, pure deionized water was used instead of mixed methanol and water. The corresponding results are presented in Figure 4.

**2.5 Equilibrium binding and competitive binding experiments with the ungrafted and grafted dual fluorescent 2,4-D-MIPs/CPs in different media**

The adsorption equilibrium performance of MIP/CP in the organic phase (methanol/water=4/1, v/v) was evaluated as follows: 6 mg samples were added to a 1.5 mL centrifuge tube. Subsequently, a mixed solution containing POAc with a concentration of 0.02 mmol/L and the template molecule 2,4-D were added to the tube at 25 °C. The mixture was shaken for 8 hours using a constant temperature shaking shaker and then centrifuged at a speed of 12000 r/min for 6 min. During this process, the polymer selectively adsorbed template molecules 2,4-D and its analogue POAc. HPLC analysis was performed to determine the amount of D and POAc present in the solution using a detection wavelength of 272 nm. A mobile phase consisting of methanol, pure water, and acetic acid (60:39.5:0.5, v/v/v) was used with a flow rate set at 1.0 mL/min. The average value from three measurements was taken for each sample while considering changes in peak area corresponding to template molecules within the solution to calculate B (μmol/g), which represents the adsorption capacity of fluorescent molecularly imprinted polymers towards both template molecule - D and its analogue POAc. The competitive adsorption performance of MIP/CP in pure water was evaluated under similar conditions as those in the organic phase; however, methanol/water mixed solvent was replaced by pure water as the solvent. The results are presented in Figure S4.

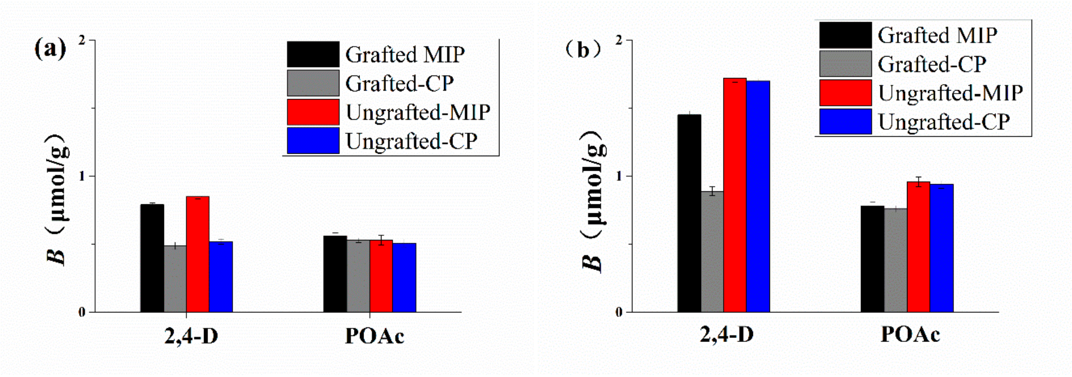


**Figure S4.** Selective adsorption results of 2,4-D and POAc mixed solution of polymer in methanol/water (4/1, v/v) (a) and pure water (b).

In order to visually demonstrate the selective adsorption ability of samples, we introduce the concept of IPB (Imprinting-induced Promotion of Binding)[1]. This parameter effectively normalizes the non-specific adsorption of different analytes in MIP, thereby characterizing its exceptional selectivity. The calculation formula for IPB is as follows:

$$IPB(\%) = [(B_{MIP} - B_{CP})/B_{CP}] \times 100\%$$

In the formula, BMIP represents the quantity of imprinted polymer MIP adsorbed on the template, while BCP denotes the quantity of non-imprinted polymer CP adsorbed on the template. The selectivity and specificity of the imprinted polymer towards the template exhibit superior performance based on IPB data, as presented in Table S1. It was evident from Table S1 that both hydrophilic molecular brushes grafted and non-grafted onto the surface of organic phase result in significantly large IPB values for imprinted polymers, indicating excellent selectivity and specific adsorption capacity without compromising imprinting efficiency. However, when tested in pure water, ungrafted imprinted polymers display negligible IPB values with a loss of selective adsorption capability; whereas hydrophilic macromolecular brush-grafted imprinted polymers still demonstrate remarkable performance by selectively adsorbing small molecule acid templates even in aqueous solutions.

**Table S1.** Calculation results of imprinted polymer's IPB values

| Solvent | Analyte | The ungrafted fluorescent 2,4-D-MIP/CP | | | The grafted fluorescent 2,4-D-MIP/CP | | |
|---|---|---|---|---|---|---|---|
| | | $B_{MIP}$[a] | $B_{CP}$[a] | IPB(%)[b] | $B_{MIP}$[a] | $B_{CP}$[a] | IPB(%)[b] |
| MeOH/$H_2O$=4:1 | 2,4-D | 0.85±0.02 | 0.52±0.02 | 63 | 0.79±0.01 | 0.49±0.03 | 62 |
| | POAc | 0.53±0.03 | 0.51±0.02 | 4 | 0.56±0.02 | 0.53±0.04 | 6 |
| $H_2O$ | 2,4-D | 1.72±0.03 | 1.70±0.02 | 1 | 1.45±0.03 | 0.89±0.03 | 62 |
| | POAc | 0.96±0.04 | 0.94±0.03 | 2 | 0.78±0.03 | 0.76±0.02 | 3 |

[a] $B_{MIP}$ and $B_{CP}$ (μmol/g) are the equilibrium binding capacities of MIP and CP toward 2,4-D and its analogues in their mixed solution ($C_{2,4\text{-D or POAc or CPOAc}}$ = 0.02 mM) in different solvent; [b] IPB refers to the "imprinting-induced promotion of binding" value of the MIP.

## 2.6 Optosensing properties of the grafted (and ungrafted) dual fluorescent 2,4-D-MIP/CP in the water

**Photostability measurements of the grafted dual fluorescent 2,4-D-MIP**

The photostability of the grafted dual fluorescent 2,4-D-MIP was evaluated by monitoring the temporal changes in fluorescence intensity of its dispersed mixture in pure water (0.25 mg/mL) at 25 °C under ambient air conditions (Figure 5).

## 2.7 Reusability of the grafted dual fluorescent 2,4-D-MIP

The reusability of the performance test was investigated by monitoring changes in fluorescence intensity before and after binding and elution of the template. The specific testing procedure was as follows: 0.5 mg of hydrophilic ratio fluorescent molecularly imprinted polymer (MIP)/cross-linked polymer (CP) composite was weighed and dispersed in 1.0 mL of deionized water, followed by measurement of its fluorescence intensity using a spectrofluorometer. After centrifugation to remove the supernatant, 1.0 mL of a 2,4-D (20 μM) aqueous solution was added, and the mixture was shaken for 1 h at 25°C on an orbital shaker before being tested again with a fluorescence spectrometer. Subsequently, centrifugation at 12000 r/min for 5 min removed any remaining liquid, and methanol was used to wash the template particles. This process was repeated ten times to evaluate the repeated use performance of the hydrophilic fluorescent MIP labeled with

ratio fluorescent probes. Changes in fluorescence intensity were measured before and after ten adsorption-desorption cycles using a fluorescence spectrophotometer (Figure 5). It can be observed that upon binding to the template molecule (2,4-D), NBD exhibited enhanced fluorescence intensity; conversely, elution resulted in decreased NBD fluorescence intensity. Throughout these ten adsorption-desorption cycles, NBD maintained consistent cyclic behavior while red quantum dots showed minimal change in their fluorescence intensities. Thus, it is demonstrated that this fluorescence sensor exhibits excellent reusability.

**2.8 Binding kinetics of the grafted dual fluorescent 2,4-D-MIP/CP**

The fluorescence kinetic study of hydrophilic ratio fluorescent MIP microparticles was conducted using the following specific test method: 0.5 mg of hydrophilic ratio fluorescent MIP/CP was accurately weighed and dispersed in a 1.0 mL aqueous solution containing 25 μM 2,4-D. The resulting mixture was vigorously shaken at a temperature of 25 °C, and the fluorescence intensity at various time points was measured utilizing fluorescence spectroscopy.

The adsorption kinetics test results of the hydrophilicity ratio fluorescently imprinted polymer microspheres are presented in Figure S5. Figures S5a and S5b illustrate the fluorescence spectra of the grafted dual fluorescent 2,4-D-MIP/CP after incubation with a 2,4-D solution at 25 °C for varying durations in an aqueous medium. Over time, there is a gradual increase in the fluorescence intensity of NBD at 500 nm, while the intensity of the red quantum dot peak at 680 nm remains relatively constant. After approximately 30 minutes, the fluorescence intensity of NBD reaches its maximum and stabilizes (Fig. S5c). Notably, MIP exhibits greater peak enhancement compared to CP, further confirming that the developed hydrophilicity ratio fluorescence possesses higher sensitivity and enables rapid detection of template molecules in aqueous solutions.

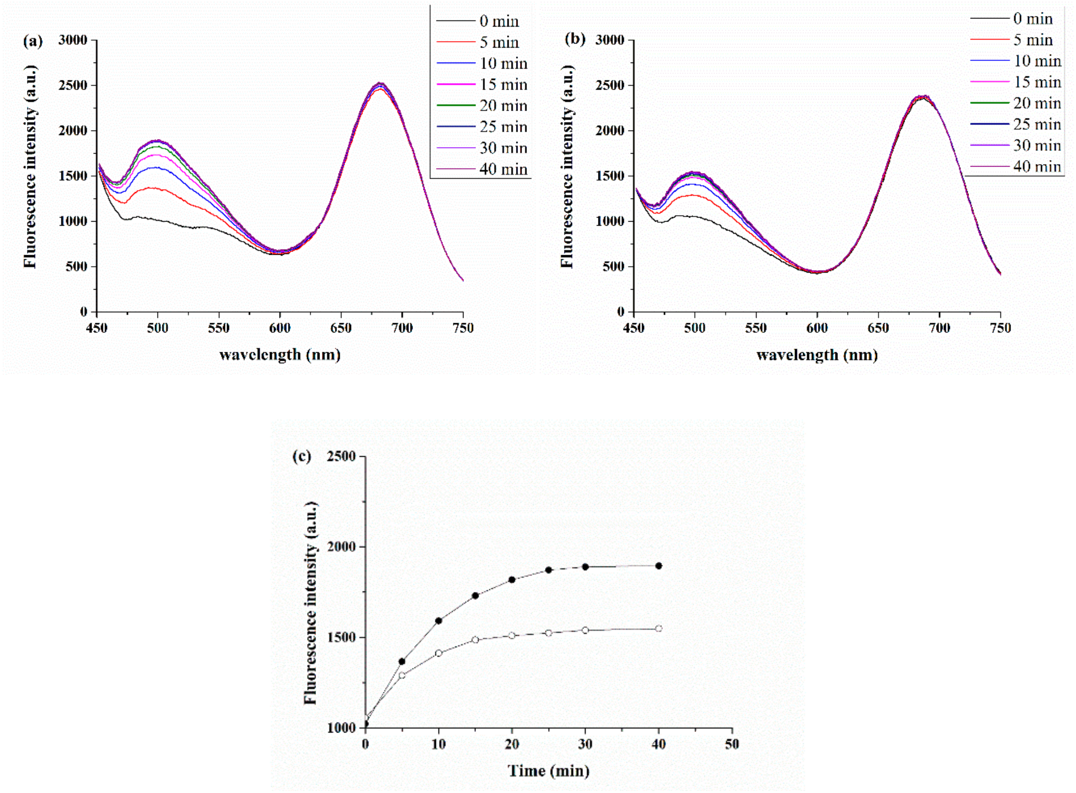


**Figure S5.** The fluorescence intensity of hydrophilic ratio fluorescent MIP/CP microspheres in 2,4-D aqueous solution over time. (a) Grafted QD, NBD-labeled 2,4-D-MIP, (b) Grafted QD, NBD-labeled 2,4-D-CP. (c) Fluorescence dynamics of hydrophilic ratio fluorescent MIP/CP microspheres.

### 2.9 Optosensing selectivity of the grafted dual fluorescent 2,4-D-MIP/CP in the aqueous solution

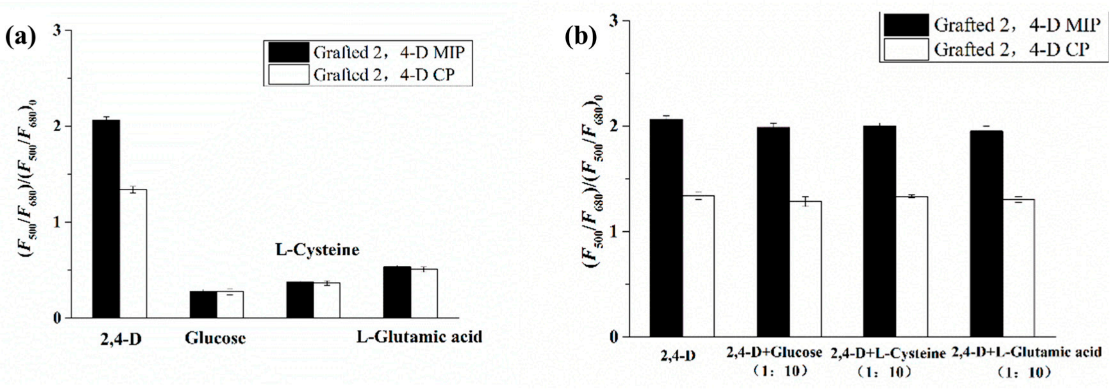


**Figure S6.** Fluorescence enhancement of the grafted dual fluorescent (filled column)/2,4-D-CP (open column) after their incubation with a solution of 2,4-D in the presence of 1 (a), and 10 (b) equivalents of mixed Glucose, L-Cysteine, and L-Glutamic acid in aqueous solution at 25 °C for 30 min.

**Table S2.** The direct quantification of 2,4-D by grafted 2,4-D MIP sensors in different real environmental aqueous solution (drinking water, lake water, urban runoff water and paddy field water) with different amounts of 2,4-D.

| Environmental water | Analyte | Concentration of analyte (μM) | | Optosensing Recovery ± RSD (%) (n = 3) |
|---|---|---|---|---|
| | | Spinked | Found | |
| paddy field water | 2,4-D | 0 | 0.15 ± 0.01 | 99.8 ± 2.3 |
| urban runoff water | 2,4-D | 0 | 0.95 ± 0.05 | 100.5 ± 1.5 |
| lake water | 2,4-D | 0 | 0 | - |
| drinking water | 2,4-D | 0 | 0 | - |
| drinking water | 2,4-D | 0.25 | 0.26 ± 0.01 | 99.2 ± 2.7 |
| drinking water | 2,4-D | 0.5 | 0. 48 ± 0.05 | 100.8 ± 1.9 |
| drinking water | 2,4-D | 1.0 | 1.02 ± 0.13 | 102.7 ± 3.3 |

[1] Xu, S.J.; Zou, Y.W.; Zhang, H.Q. Well-defined hydrophilic "turn-on"-type ratiometric fluorescent molecularly imprinted polymer microspheres for direct and highly selective herbicide optosensing in the undiluted pure milks. Talanta 2020, 211, 120711.
